# Genetic Basis of Carnivorous Leaf Development

**DOI:** 10.3389/fpls.2021.825289

**Published:** 2022-01-13

**Authors:** Arpita Agrawal, Ashwani Pareek, Jeremy Dkhar

**Affiliations:** ^1^Plant EvoDevo Laboratory, Agrotechnology Division, CSIR-Institute of Himalayan Bioresource Technology, Palampur, India; ^2^Stress Physiology and Molecular Biology Laboratory, School of Life Sciences, Jawaharlal Nehru University, New Delhi, India; ^3^National Agri-Food Biotechnology Institute, Mohali, India; ^4^Academy of Scientific and Innovative Research, Ghaziabad, India

**Keywords:** carnivorous plants, leaf development, *Nepenthes*, *Utricularia*, *Sarracenia*

## Abstract

Plant carnivory is often manifested as dramatic changes in the structure and morphology of the leaf. These changes appear to begin early in leaf development. For example, the development of the *Sarracenia purpurea* leaf primordium is associated with the formation of an adaxial ridge, whose growth along with that of the leaf margin resulted in a hollow structure that later developed into a pitcher. In *Nepenthes khasiana*, pitcher formation occurs during the initial stages of leaf development, although this has not been shown at the primordial stage. The formation of the *Utricularia gibba* trap resulted from the growth of the dome-shaped primordium in both the longitudinal and transverse directions. Recent research has begun to unfold the genetic basis of the development of the carnivorous leaf. We review these findings and discuss them in relation to the flat-shaped leaves of the model plant *Arabidopsis*.

## Introduction

**FIGURE 1:**
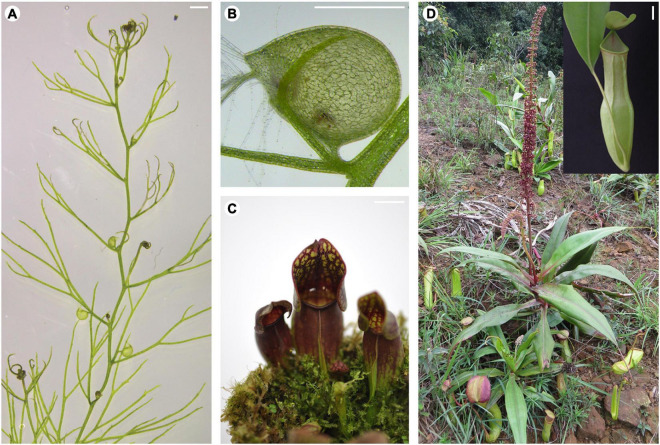
Innovative leaf morphologies of selected carnivorous plants. **(A)** Stolon of a *U. gibba* plant showing leaves with or without traps. **(B)** Close-up view of a *U. gibba* trap. **(C)** Pitcher-shaped leaves of *S. purpurea*. **(D)** A *N. khasiana* plant in the wild bearing pitcher-shaped leaves (inset shows close-up view of the *N. khasiana* pitcher). **(A,B)** Bar = 1 mm; **(C,D)** bar = 10 mm.

**FIGURE 2:**
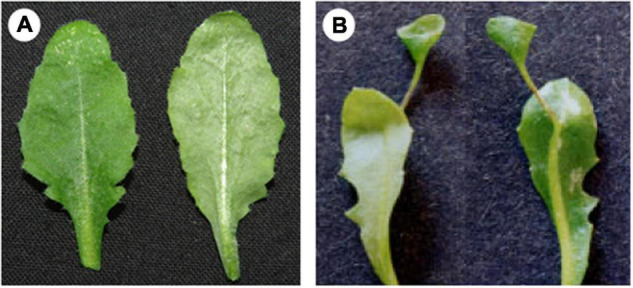
Leaf phenotypes of wild-type and mutant *Arabidopsis* plants. **(A)** Flat-shaped leaves of wild-type *Arabidopsis* plant. **(B)** Trumpet-shaped leaves of *rev* gain-of-function mutant *Arabidopsis* plant. Note the resemblance of the mutant leaf with those of *Nepenthes* (inset in Figure 1D). Images on the left and right of **(A,B)** correspond to the adaxial and abaxial sides, respectively. **(B)** Is reproduced from Zhong and Ye (2004) with permission from Oxford University Press, United Kingdom.

Carnivorous plants develop a set of morphological features termed “carnivorous syndrome”, which facilitate the capture and digestion of attracted prey (Pavlovič et al., 2007). This syndrome is manifested mostly on the leaves – as innovative morphological structures (Figure 1) – and has been the focus of research ever since the publication of Charles Darwin’s book *Insectivorous plants* (Darwin, 1875; Ellison and Gotelli, 2009). Studies have now shed light on why plant carnivory evolved, leading to a deeper understanding of the underlying mechanisms governing prey entrapment and digestion. It took a 140 years later, through an excellent study involving the purple pitcher plant *S. purpurea* (Fukushima et al., 2015), that the developmental basis of these highly specialized leaves became known. A few years later, the developing leaf transcriptome of *N. khasiana* was reported, hinting at the possible link between leaf polarity genes and pitcher formation (Dkhar and Pareek, 2019). Another outstanding study from the Coen lab provided insights on the formation of the *U. gibba* trap (Whitewoods et al., 2020). These studies have now begun to reveal the molecular mechanisms underpinning the development of the carnivorous leaf, shaped either as a cup (in *U. gibba*) or a pitcher (in *S. purpurea*). These innovative leaf morphologies appear to have evolved independently through changes in the existing genetic mechanisms governing flat-shaped leaf development (e.g., *Arabidopsis*, Figure 2A) (Lee et al., 2019).

## Leaf Organogenesis at the Shoot Apical Meristem of Carnivorous Plants

Leaves arise from the peripheral zone of the SAM (Bowman and Floyd, 2008). In *Arabidopsis* and other model plant organisms, the process begins with the recruitment of leaf founder cells (Dkhar and Pareek, 2014). In carnivorous plants, initiation of the leaf primordium might also involve founder cells recruitment. This recruitment is critical to determine the region at the flanks of SAM where primordia will initiate. Initiation is then followed by the establishment of the adaxial/abaxial polarity along with the bulging of the leaf primordia (Tsukaya, 2013). The developmental processes leading to the protrusion of the incipient leaf primordia appear to be common among land plants. As development progresses, structural changes begin to appear on the leaf primordia of carnivorous plants. In *S. purpurea*, the adaxial side of the developing leaf primordium becomes elevated, forming a ridge (Fukushima et al., 2015). Further growth and development of the adaxial ridge resulted in the formation of a hollow structure at the distal part of the primordium, which eventually develops into a pitcher (Fukushima et al., 2015). In *Arabidopsis*, the developing leaf primordium remains flat until maturity (Matsumoto and Okada, 2001) (Figure 2A). In *U. gibba*, trap develops laterally on a leaf. But not all *U. gibba* leaf bears a trap; rather, filiform-shaped leaflets develop. What specifies a *U. gibba* leaf to develop a trap instead of a leaflet is inherently genetic (discussed below). Interestingly, both leaflet and trap primordia look alike during the initial stages of development (Whitewoods et al., 2020). Changes appear at later stages: leaflet primordia turn into cylinders with tapered ends whereas trap primordia become spherical in shape, due to growth in both the longitudinal and transverse directions (Whitewoods et al., 2020). In *Nepenthes*, pitchers are attached to a flattened leaf base lamina *via* a tendril. It was previously thought that pitcher initiation occurs at the tip of a tendril (Owen and Lennon, 1999). However, recent evidence suggests that pitcher formation in *N. khasiana* occurs early in leaf development and shares anatomical features with the young in-rolled leaf base lamina (Dkhar and Pareek, 2019). It remains to be seen though, how pitcher initiation occurs on the leaf primordium of *Nepenthes*.

## Trap Morphogenesis in *Utricularia gibba* Is a Result of Restricted Gene Expression

How does a *U. gibba* plant direct its leaf to develop a trap, instead of a leaflet? The answer lies in the expression pattern of the *PHAVOLUTA* (*PHV*) gene, a member of the class III *HD-ZIP* gene family known to specify the adaxial identity of lateral organs (McConnell et al., 2001). The *Arabidopsis* genome contains five *HD-ZIPIII* genes namely *PHABOLUSA* (*PHB*), *REVOLUTA* (*REV*), *ATHB8*, *ATHB15*, and *PHV* (Emery et al., 2003). In *U. gibba*, *PHV* showed extended as well as restricted expression patterns on the adaxial side of the developing leaf primordia (Whitewoods et al., 2020). This differential gene expression pattern carries biological significance: primordia showing restricted *PHV* expression developed into traps. When this restricted expression pattern is perturbed, through heat-shock-induced ectopic expression of *PHV* that is altered to prevent recognition by miRNA (preferably miR165), allowing *PHV* expression throughout the leaf tissue, trap development is significantly reduced (Whitewoods et al., 2020). Thus, trap initiation in *U. gibba* is a result of restricted *PHV* expression. In *S. purpurea*, however, no distinct *PHB* or *FIL* (*FILAMENTOUS FLOWER*, a gene specifying abaxial identity) expression patterns were seen between the hollow and the ridge regions, indicating that neither of the two genes play a role in pitcher initiation. Instead, changes in the orientation of cell division in the developing leaf primordium led to the development of the pitcher-shaped leaf in *S. purpurea* (Fukushima et al., 2015).

## *Arabidopsis REVOLUTA* Mutants Display *Nepenthes* Leaf Phenotype

*Arabidopsis* mutants of the *HD-ZIPIII* genes viz. *PHB*, *PHV* and *REV* display leaf phenotypes similar to those seen in pitcher plants, particularly *Nepenthes*. These gain-of-function mutations transform the flat-shaped leaves of *Arabidopsis* into trumpet-shaped (McConnell and Barton, 1998; McConnell et al., 2001; Emery et al., 2003; Zhong and Ye, 2004). In severe cases, these trumpet-shaped leaves grew out from the abaxial midvein of the slightly narrower leaf lamina (Figure 2B), resembling the pitcher-shaped leaves of *Nepenthes*. Unlike *phb* and *phv* gain-of-function mutants, the inside surface of the trumpet-shaped leaf of gain-of-function *rev* mutants is adaxial, as is the case with *Nepenthes* pitchers. The *rev* phenotype is due to a single nucleotide change in the putative lipid/sterol-binding START domain of the *REV* gene, which prevented recognition and thereby negative regulation by miR165 resulting in the expanded expression of *REV* (Emery et al., 2003). In *N. khasiana*, *REV* showed increased expression in the tip of the young *N. khasiana* leaf that later developed into a pitcher (Dkhar and Pareek, 2019). We now know that increased and restricted expression pattern of *PHV* resulted in trap formation in *U. gibba*. It is likely then that the increased and expanded expression of *REV* probably led to the development of the *Nepenthes* pitcher.

## Auxin and Its Role in Pitcher Development

**FIGURE 3:**
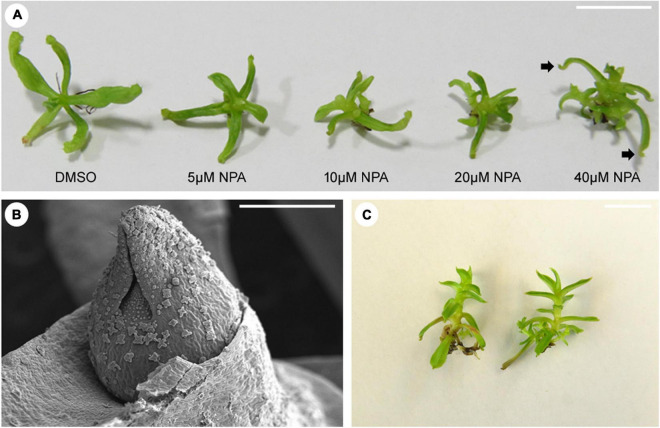
Effects of auxin transport inhibition and nutrient availability on pitcher formation in *N. khasiana*. **(A)** Morphological phenotypes of *N. khasiana* plantlets after 1 month of treatment with varying concentrations of NPA (arrow points to tiny structures at the tip of the leaf). **(B)** A scanning electron micrograph of an axillary bud of *N. khasiana*, arising as a result of NPA treatment. **(C)** *N. khasiana* plantlets grown in 2 MS (twice the strength of full MS i.e., 1 MS). Note the dramatic changes in leaf morphology. **(A,C)** Bar = 10 mm; **(B)** bar = 0.5 mm.

Trumpet-shaped leaves were also seen in *Arabidopsis* plants with defective *PIN1* and *REV* genes, but not on single *pin1* or *rev Arabidopsis* mutants (Qi et al., 2014). *PIN1* encodes for an auxin efflux carrier protein that mediates local auxin accumulation during leaf initiation (Reinhardt et al., 2003). Based on their observations, Qi et al. (2014) suggested that auxin and *REV* act independently to promote leaf polarity. Fukushima et al. (2015) evaluated the effects of auxin on pitcher development in *S. purpurea*. Neither the addition of 1-Naphthaleneacetic acid (NAA) nor 1-N-Naphthylphthalamic acid (NPA), an auxin transport inhibitor, prevented the formation of the ridge and the hollow regions in the leaf primordia of *S. purpurea*. In *N. khasiana*, addition of NPA at 5, 10, 20, and 40 μM concentrations to the growing 1/4 strength MS medium increases shoot branching, as a result of axillary bud growth (Figures 3A,B). At higher NPA concentration, pitchers of *N. khasiana* failed to develop, although a tiny structure at the tip of the leaf can still be seen (Figure 3A). Altogether, these results suggest that auxin might have an indirect role in the formation (or the expansion) of the *N. khasiana* pitcher.

## Temperature and Nutrient Availability Affect Pitcher Formation

Leaf shape can vary to change in environmental conditions such as temperature and light (Dkhar and Pareek, 2014). Interestingly, carnivorous plants also respond to change in temperature and nutrient availability. When the Australian pitcher plant *Cephalotus follicularis* was grown at 15°C under continuous light conditions, more flat leaves developed than pitcher-shaped leaves (Fukushima et al., 2017). At 25°C, however, the reverse was observed. Could this be a result of the plant’s response to higher temperatures? We know that *Nepenthes*, including *N. khasiana*, grow in nutrient-deficient soil. So, upon adding sufficient nutrients to the substratum, prey-deprived *N. talangensis* plants respond to this change in condition by developing leaves that lack pitchers (Pavlovič et al., 2010). We replicated this experiment *in vitro* by growing dissected shoot apices of 3-months old *N. khasiana* seedlings in MS medium of varying strengths (Dkhar et al., unpublished data). Most shoot apices of *N. khasiana* grown in 2 MS (twice the strength of full MS i.e., 1 MS) failed to grow, but if they survive, the plantlets develop narrower leaves completely devoid of pitchers (Figure 3C). By modifying nutrient content in the growing medium, we are now presented with *N. khasiana* plants lacking pitchers. It would be interesting to know how nutrient availability signals the plant to develop or not to develop pitchers.

## Conclusion

Carnivorous plants evolved pitcher- or cup-shaped leaves independently in four angiosperm lineages viz. Cephalotaceae (*Cephalotus*), Lentibulariaceae (*Utricularia*), Nepenthaceae (*Nepenthes*) and Sarraceniaceae (*Sarracenia*) (Whitewoods and Coen, 2017). Although these leaves may look complex in shape and form, research suggests that their formation may be a result of simple modification – through changes in gene expression patterns and growth modulation – of existing leaf developmental programs at work in simpler leaf forms. Studies on the model plant *Arabidopsis* have led to a deeper understanding of the underlying mechanisms controlling leaf development, which now form the basis of ongoing research in the study of the evolution and development of innovative leaf morphologies seen across angiosperms, including carnivorous plants. The tremendous progress seen in *Arabidopsis* is, in part, attributed to the ease at which this plant can be genetically transformed, allowing various genes for leaf development to be functionally validated. Recent progress in this direction, such as the establishment of *Agrobacterium*-mediated transformation protocols in *N. mirabilis* (Miguel et al., 2020) and *U. gibba* (Oropeza-Aburto et al., 2020), offers much-needed hope for a complete genetic dissection of these complex leaf shapes.

## Author Contributions

JD conceived the review. AA wrote the initial manuscript, which was then corrected and finalized by JD. AP did the final editing. All authors read and agreed with the final content of the manuscript.

## Conflict of Interest

The authors declare that the research was conducted in the absence of any commercial or financial relationships that could be construed as a potential conflict of interest.

## Publisher’s Note

All claims expressed in this article are solely those of the authors and do not necessarily represent those of their affiliated organizations, or those of the publisher, the editors and the reviewers. Any product that may be evaluated in this article, or claim that may be made by its manufacturer, is not guaranteed or endorsed by the publisher.
